# Growth of Zn–N Co-Doped Ga_2_O_3_ Films by a New Scheme with Enhanced Optical Properties

**DOI:** 10.3390/nano15131020

**Published:** 2025-07-01

**Authors:** Daogui Liao, Yijun Zhang, Ruikang Wang, Tianyi Yan, Chao Li, He Tian, Hong Wang, Zuo-Guang Ye, Wei Ren, Gang Niu

**Affiliations:** 1 State Key Laboratory for Manufacturing Systems Engineering, Electronic Materials Research Laboratory, Key Laboratory of the Ministry of Education, School of Electronic Science and Engineering, Xi’an Jiaotong University, Xi’an 710049, China; 2 Instrumental Analysis Center, Xi’an Jiaotong University, Xi’an 710049, China; 3 Department of Chemistry, 4D LABS, Simon Fraser University, Burnaby, BC V5A 1S6, Canada; 4 State Key Laboratory for Manufacturing Systems Engineering & The International Joint Laboratory for Micro/Nano Manufacturing and Measurement Technology, Xi’an Jiaotong University, Xi’an 710049, China

**Keywords:** gallium oxide, atomic layer deposition, Zn–N co-doping, optoelectronic properties

## Abstract

Gallium oxide (Ga_2_O_3_), as a wide-bandgap semiconductor material, is highly expected to find extensive applications in optoelectronic devices, high-power electronics, gas sensors, etc. However, the photoelectric properties of Ga_2_O_3_ still need to be improved before its devices become commercially viable. As is well known, doping is an effective method to modulate the various properties of semiconductor materials. In this study, Zn–N co-doped Ga_2_O_3_ films with various doping concentrations were grown in situ on sapphire substrates by atomic layer deposition (ALD) at 250 °C, followed by post-annealing at 900 °C. The post-annealed undoped Ga_2_O_3_ film showed a highly preferential orientation, whereas with the increase in Zn doping concentration, the preferential orientation of Ga_2_O_3_ films was deteriorated, turning it into an amorphous state. The surface roughness of the Ga_2_O_3_ thin films is largely affected by doping. As a result of post-annealing, the bandgaps of the Ga_2_O_3_ films can be modulated from 4.69 eV to 5.41 eV by controlling the Zn–N co-doping concentrations. When deposited under optimum conditions, high-quality Zn–N co-doped Ga_2_O_3_ films showed higher transmittance, a larger bandgap, and fewer defects compared with undoped ones.

## 1. Introduction

Gallium oxide (Ga_2_O_3_) has been considered for a wide spectrum of applications in transparent conducting oxides, deep ultraviolet photodetectors, and high-voltage power electronic devices [1,2,3,4,5], thanks to its fascinating features, such as an ultra-wide bandgap of 4.9 eV [6,7], a high breakdown voltage (8 MV/cm) [8,9], and a high Baliga’s figure of merit (BFOM) of around 3444 [10,11]. However, due to the facile presence of oxygen vacancies, Ga_2_O_3_ generally displays n-type semiconducting properties [12], which limits its practical applications.

Doping with selective elements is an effective method to control the electronic and optical properties of semiconductors, including Ga_2_O_3_. In recent years, a series of Ga_2_O_3_-based materials with excellent properties have been successfully prepared based on this strategy [13,14]. The conductivity of Ga_2_O_3_ can be adjusted by doping with Sn, Ge, or Si elements [15,16,17,18], and the optical bandgap of Ga_2_O_3_ semiconductors can be tuned by doping with Zn and Al [19,20]. Great efforts have been made to improve the results of p-type doping of Ga_2_O_3_-based materials [19,21,22,23]. Given the similar ionic radii of N^3−^ and O^2−^, nitrogen is considered one of the most promising dopants for regulating the electronic and optical properties of Ga_2_O_3_-based materials. However, effective high-concentration nitrogen doping is usually difficult to achieve in most studies. Notably, high-quality nitrogen-doped p-type Ga_2_O_3_ materials were successfully prepared by the thermal oxidation of gallium nitride (GaN) on sapphire substrates at 1000–1100 °C [24,25]. First-principles calculations showed that tunable optical properties and even p-type conductivity can be achieved through co-doping with Zn and N. However, it is very difficult to achieve co-doping in the experiment. So far, the influence of Zn and N co-doping on the physical properties of Ga_2_O_3_ films is still unknown experimentally. Therefore, it is rewarding to take on this challenge in this work.

Several deposition techniques have been used to prepare Ga_2_O_3_ films, such as molecular beam epitaxy (MBE) [26,27], pulsed laser deposition (PLD) [28,29], magnetron sputtering, metal–organic chemical vapor deposition (MOCVD) [30], and atomic layer deposition (ALD) [31,32]. Compared with other thin film deposition techniques, ALD offers many advantages, such as precise thickness control, excellent 3D conformality, wafer-scale uniformity, compatibility with the mass production of semiconductor integrated circuits, etc. These advantages position ALD among the most promising ultra-thin and conformal thin film growth technologies, enabling precise dopant concentration control. However, the low deposition temperature characteristic of ALD has led to amorphous or polycrystalline Ga_2_O_3_ films. Furthermore, systematic investigations into controlled doping—particularly co-doping—of Ga_2_O_3_ via ALD remain scarce, hindering the mechanistic understanding of dopant effects in these thin films. In this work, the Zn–N co-doped β-Ga_2_O_3_ films were achieved by ALD and the effects of the doping concentrations of Zn and N elements on the crystallinity, optical bandgap, surface morphology, and photoluminescence properties were systematically investigated. GaN/ZnO nano-laminated films were grown by combining the plasma-enhanced ALD (PEALD) of GaN and the thermal ALD of ZnO at 250 °C, and then the Zn–N co-doped Ga_2_O_3_ films were prepared by the thermal oxidation of the deposited GaN/ZnO films at 900 °C. The Zn–N co-doping concentration was tuned by adjusting the ratio of the ALD growth cycles of the GaN and ZnO monolayers.

## 2. Experimental Procedures

### 2.1. Sample Preparation

The depositions of the GaN/ZnO nano-laminated films were conducted on c-plane (0001) sapphire (alpha-Al_2_O_3_) wafers at 250 °C in an ALD (R200, Picosun, Espoo, Finland) reactor. Prior to the reactions, the wafers were sequentially rinsed with acetone, ethyl alcohol, and deionized (DI) water in an ultrasonicator for 10 min, and then dried with a N_2_ gun before being placed into the reaction chamber. The Zn–N co-doped Ga_2_O_3_ films were fabricated by the thermal oxidation of the ALD-deposited GaN/ZnO nano-laminated films. GaN was grown using triethylgallium (TEGa) and an Ar/N_2_/H_2_ (1:3:6) mixture gas plasma as the Ga and N precursors, respectively. ZnO was grown from the precursor of DI water and diethylzinc (DEZn) through thermal ALD. The precursor of TEGa was heated up to 60 °C, while the DI water and DEZn precursor was kept at room temperature (about 20 °C). N_2_ (99.999%) was utilized as the carrier gas in the experiment. During the remote plasma process, the mixture plasma gas flow and RF power were set to 180 sccm and 2000 W, respectively. The PEALD growth sequence of GaN consisted of TEGa exposure (0.1 s), N_2_ purging (5 s), Ar/N_2_/H_2_ plasma exposure (12 s), and N_2_ purging (4 s). The thermal ALD growth sequence of ZnO consisted of DEZn exposure (0.1 s), N_2_ purging (5 s), DI water exposure (0.1 s), and N_2_ purging (5 s). After several cycles (*n* = 12, 6, or 3) of GaN deposition, 1 ZnO cycle was performed to complete a supercycle of Zn-doped GaN deposition. The Zn-doped GaN films were deposited after several supercycles. The details of the film composition are provided in Table 1. In order to achieve Zn–N co-doped Ga_2_O_3_ and to activate the doping atoms, the as-grown Zn-doped GaN films were oxidized through post-annealing at 900 °C for 1 h, followed by naturally cooling down to room temperature. The resulting Zn–N co-doped Ga_2_O_3_ films are named as N-Ga(n)Zn(1), where n denotes the number of cycles of GaN in the supercycle of Zn-doped GaN deposition. For comparison, the GaN films without Zn doping were deposited after 500 cycles of GaN. The GaN films were transformed into N-doped Ga_2_O_3_ (N-Ga_2_O_3_) film by post-annealing at 900 °C in air. Furthermore, for comparison, a pure Ga_2_O_3_ film was deposited in situ using the following process parameters: TEG exposure (0.1 s), N_2_ purging (6 s), O_2_ plasma exposure (10 s), and N_2_ purging (5 s).

### 2.2. Characterization

X-ray diffraction was employed to analyze the crystalline quality of the films (XRD, Bruker, Bremen, Germany) using Cu Kα radiation (45 kV, 40 mA, λ = 1.54056 Å) in the 2θ range of 20 to 120°. The morphology of the thin films was examined using atomic force microscopy (AFM, Bruker Dimension Icon, Bremen, Germany) in tapping mode. An ultraviolet–visible spectrophotometer was used to measure the absorption spectra of the thin films in the wavelength range of 190–800 nm. The Tauc plots based on the UV–vis data were further used to determine the optical bandgap. The microstructures of the thin films were investigated via transmission electron microscopy (TEM, JEOL JEM-F200, Tokyo, Japan). A secondary ion mass spectrometer (SIMS, Hesse, Germany) was employed to investigate the elemental distribution and diffusion in the films. The photoluminescence (PL) performance of the samples was evaluated by fluorescence spectrometry (FLS1000, Techcomp, Edinburgh, UK).

## 3. Results and Discussion

**Figure 1:**
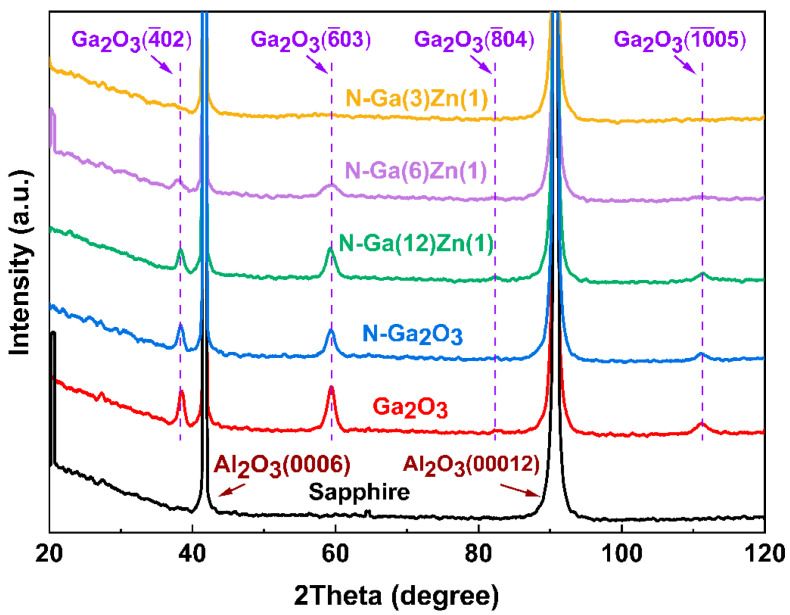
XRD patterns of the Zn–N co-doped Ga_2_O_3_ films with different doping concentrations and the undoped Ga_2_O_3_ thin films after post-annealing at 900 °C.

Figure 1 shows the XRD patterns of the Zn–N co-doped Ga_2_O_3_ films post-annealed at 900 °C with various doping concentrations. The undoped Ga_2_O_3_ film post-annealed at 900 °C shows four significant diffraction peaks, corresponding to the $\left(\bar{4}02\right)$, $\left(\bar{6}03\right)$, $\left(\bar{8}04\right)$, and $\left(\bar{10}05\right)$ peaks of β-Ga_2_O_3_. The N-doped Ga_2_O_3_ film (N-Ga_2_O_3_) exhibits a similar XRD diffraction pattern to the undoped Ga_2_O_3_ film, indicating that the pure GaN films are oxidized into N-doped Ga_2_O_3_ through post-annealing at 900 °C, and the nitrogen doping has little effect on the lattice structure and crystallinity of the Ga_2_O_3_ films. The above results reveal that both undoped and N-doped Ga_2_O_3_ films show a preferential orientation. However, with the increase in the doping concentration of the Zn element, the intensity of the X-ray diffraction peaks of the Ga_2_O_3_ films becomes weaker or even disappears, showing that Zn doping deteriorates the crystallinity of the Ga_2_O_3_ films. The degradation of the crystal structure at high Zn doping levels may be partly attributed to insufficient oxidation kinetics during the post-annealing of Zn-enriched GaN/ZnO nanolayers, resulting from inadequate oxygen partial pressure and annealing time at elevated temperatures.

**Figure 2:**
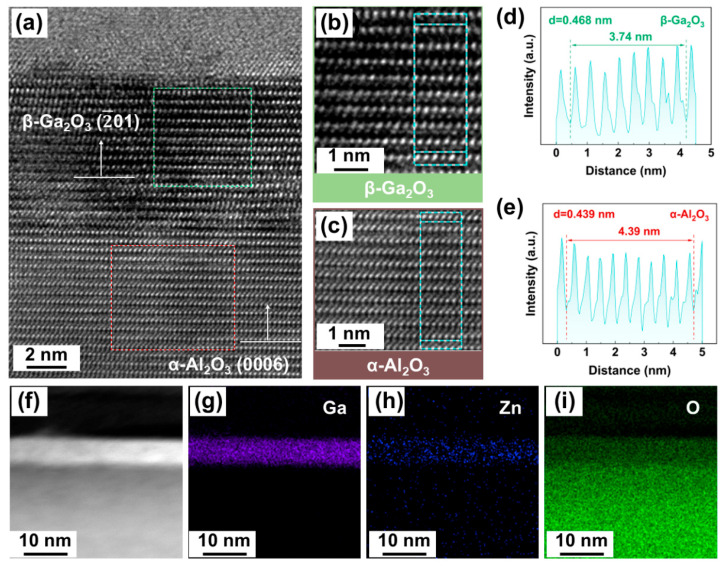
(**a**–**c**) are the HRTEM images of the N-Ga(6)Zn(1) Ga_2_O_3_ film. (**d**,**e**) are the intensity profiles corresponding to the green rectangular marked regions in (**b**,**c**). (**f**–**i**) are the TEM images (**f**) and the corresponding EDS mappings of the Ga (**g**), Zn (**h**), and O (**i**) elements.

The detailed microstructure of the post-annealed N-Ga(6)Zn(1) Ga_2_O_3_ film was analyzed using FE-TEM and the results are shown in Figure 2. As shown in Figure 2a–e, the films have an atomically sharp interface, and no buffer or nucleation layer is observed at the interface of Ga_2_O_3_/Al_2_O_3_. A well-aligned crystal lattice image can be seen in the Zn–N co-doped film of Ga_2_O_3_, without any metastable phases observed. The high-resolution TEM (HRTEM) images show a lattice spacing of 0.469 nm for Ga_2_O_3_, corresponding to the $\left(\bar{2}01\right)$ interplanar distance of the β-Ga_2_O_3_ structure. Combining the in-plane results of HRTEM and the out-of-plane results of XRD, the growth relationship can be summarized as β-Ga_2_O_3_ $\left(\bar{2}01\right)$//α-Al_2_O_3_ (0001). Since the crystal planes of the β-Ga_2_O_3_ $\left(\bar{2}01\right)$ family are polar planes (i.e., consisting of only one type of atom, either Ga or O), it can be deduced that the self-limited alternating reactions process of ALD favors the formation of polar planes in the thin films [31]. The low magnification cross-sectional TEM imaging and the corresponding EDS mapping analyses were further carried out on the N-Ga(6)Zn(1) Ga_2_O_3_ film. As shown in Figure 2b–e, the EDS mapping of Ga reveals a strong and sharp contrast in the Ga_2_O_3_ film, while the contrast for the Zn element is obviously weak and uniformly distributed in the Ga_2_O_3_ film. Moreover, no evident diffusion of Ga into the sapphire substrate was observed, indicating that no solid reaction or interdiffusion occurred between Ga_2_O_3_ and Al_2_O_3_ during the high-temperature post-annealing process at 900 °C. The concentration of the oxygen element in the film edge is lower than in the substrate, which may be due to the presence of oxygen vacancies and the substitution of the N element. Because the doping concentration of nitrogen is very low and the nitrogen in the air is physically adsorbed onto the surface of the TEM sample, it is difficult to identify any N element present in the films by the EDS mapping results.

**Figure 3:**
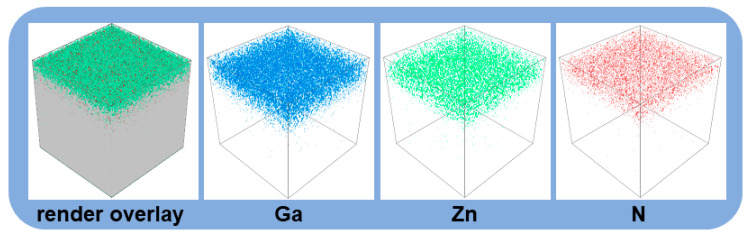
SIMS elemental mapping of Ga (blue), Zn (green), and N (red) in the post-annealed N-Ga(12)Zn(1) film.

In order to confirm that nitrogen is indeed successfully incorporated into the Ga_2_O_3_ films, the vertical distribution of the N, Zn, and Ga elements in the films were further analyzed using SIMS. Figure 3 displays the SIMS results of the post-annealed N-Ga(12)Zn(1) film, where the presence of the Ga (blue), Zn (green), and N (red) elements are clearly observed by the SIMS elemental mapping, confirming the successful co-doping of Zn and N elements in the Ga_2_O_3_ film. It is worth noting that the N and Zn doping elements are uniformly distributed throughout the entire Ga_2_O_3_ film, indicating that the doping is very uniform.

**Figure 4:**
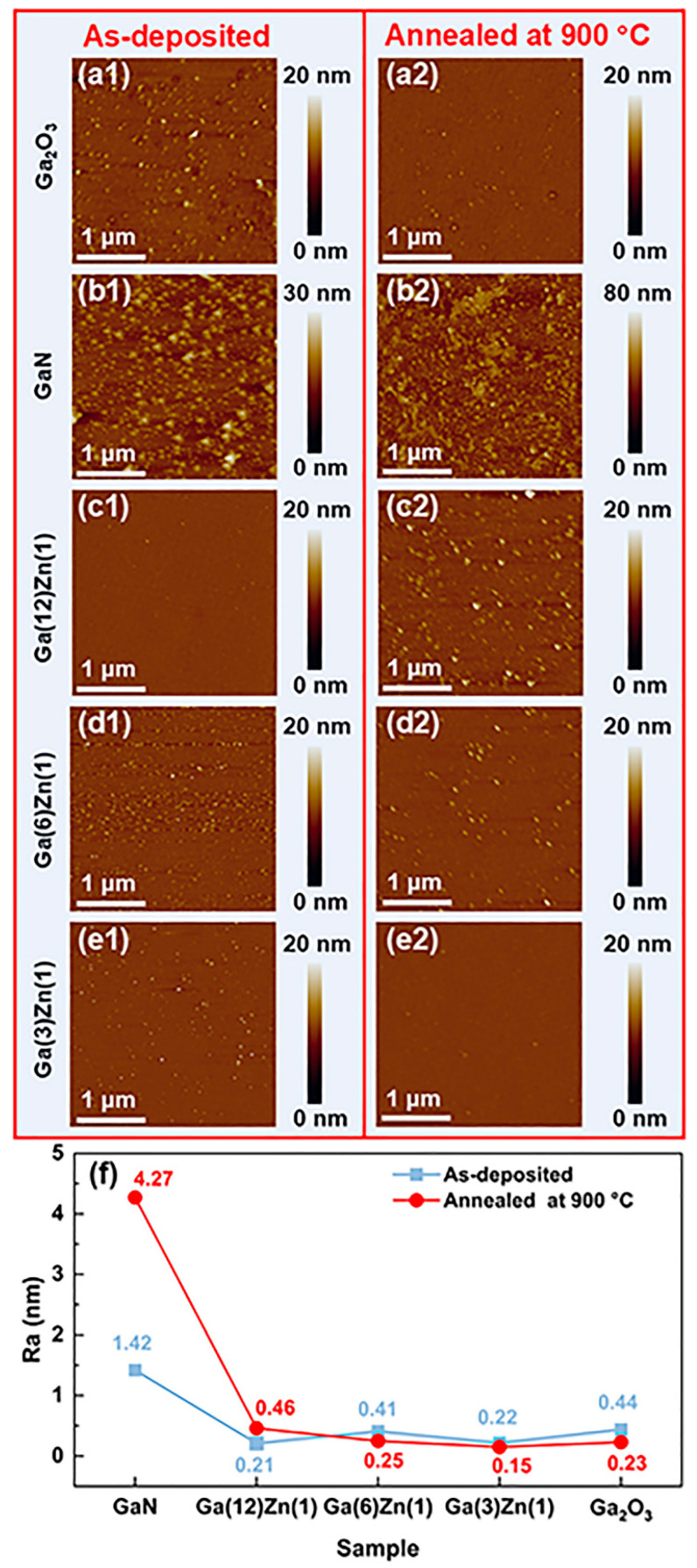
(**a1**–**e2**) AFM images of the as-deposited films (**a1**,**b1**,**c1**,**d1**,**e1**) and the corresponding post-annealed films (**a2**,**b2**,**c2**,**d2**,**e2**). (**f**) Average roughness of the as-deposited and post-annealed films.

Figure 4 shows the AFM images and the root mean square (RMS) average roughness of various samples. The surface morphology of the as-deposited pure GaN film exhibits a large number of dispersed particles, as seen in Figure 4(b1). Compared with the undoped GaN films, the surface morphology of the Zn-doped GaN films reveals much less or no gross grains (Figure 4(c1–e1)), with a much-reduced average roughness (Ra), indicating that Zn doping can improve the surface smoothness of the GaN films. As seen in Figure 4(a1), the in situ grown Ga_2_O_3_ film exhibits a comparable morphology quality with the Zn-doped GaN samples. After post-annealing at 900 °C, the pure GaN and Zn-doped GaN films transformed into the N-doped Ga_2_O_3_ thin film and Zn–N co-doped Ga_2_O_3_ thin films, respectively, and their AFM surface morphology images are shown in Figure 4(b2–e2). As shown in Figure 4f, the roughness of the Zn-doped GaN films is almost the same as the undoped Ga_2_O_3_ film. However, the average roughness of the undoped GaN film increases from 1.42 nm to 4.27 nm after post-annealing at 900 °C, with the particles merged together to form larger aggregates, as presented in Figure 4(b2). This could be attributed to the oxidation and the transformation from an amorphous to a crystalline state. The surface roughness of the Zn–N co-doped Ga_2_O_3_ films monotonically decreases with the increase in Zn doping concentration. More importantly, the surface morphology of the N-Ga(3)Zn(1) film is smoother than the undoped Ga_2_O_3_ film, which may be attributed to the amorphous microstructure as confirmed by the XRD results.

**Figure 5:**
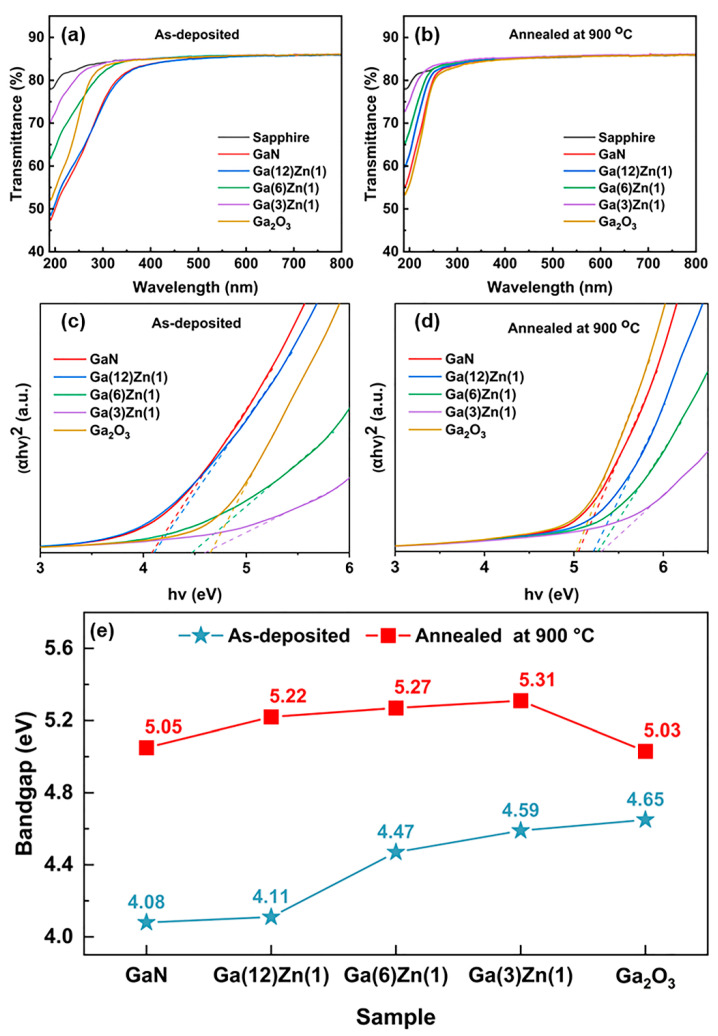
Optical transmittance spectra of the as-deposited films (**a**) and after post-annealing at 900 °C films (**b**). Tauc plots of the as-deposited films (**c**) and the films post-annealed at 900 °C (**d**), with the optical bandgaps shown on the horizontal axis by extrapolation of the curves. The optical bandgaps of the films obtained from the Tauc plots (**e**).

In order to characterize the optical properties and bandgap of the films, optical transmittance was measured using UV–vis spectroscopy in the wavelength region from 190 nm to 800 nm. As shown in Figure 5a, all of the as-deposited films exhibit outstanding optical transmittance in the visible wavelength range from 400 nm to 780 nm. The transmittance of all of the as-deposited films drops sharply in the UV spectral range of 300 to 190 nm. The transmittance of the as-grown GaN film is the lowest, indicating the strongest absorption of UV light. The as-grown N-Ga(12)Zn(1) film exhibits a similar transmittance to the as-grown GaN film, owing to the close chemical composition between them. The UV spectral transmittance increases noticeably when the Zn concentration increases, indicating that the optical bandgap increases with the increasing concentration of Zn. After post-annealing at 900 °C, the UV region transmittances of all of the films are improved and the absorption edges are obviously blue shifted. At the same time, the N-doped Ga_2_O_3_ film (transformed from the as-grown GaN film) has a similar transparency to the undoped Ga_2_O_3_ film, which further indicates that the GaN film has been successfully oxidized into N-doped Ga_2_O_3_ films. More importantly, the increase in the doping concentrations of Zn elements in the Zn–N co-doped Ga_2_O_3_ films causes the absorption band edge of the co-doped Ga_2_O_3_ to obviously blue shift, which is due to the rise in carrier concentration [33].

For a direct bandgap semiconductor material, we can obtain the optical bandgap (E_g_) of the films by extrapolating the linear part of the Tauc plot to zero. The Tauc plot is the plot of ${\left(\alpha hv\right)}^{2}$ versus hυ, and it can be described by the following equation:

(1) $${\left(\alpha hv\right)}^{2}\propto B(hv-E_{g}),$$

where h is the Planck’s constant, α is the absorption coefficient, ν is the frequency of the incident light, B is a constant, and E_g_ is the bandgap energy.

As shown in Figure 5e, before post-annealing at 900 °C, the undoped Ga_2_O_3_ film has the largest bandgap of 4.69 eV, consistent with the previous reports [34]. It is worth noting that the bandgap of the deposited GaN film is 4.08 eV, higher than the reported 3.4 eV, which could be due to the presence of oxygen impurities [35]. The bandgap increases with the increase in ZnO/GaN deposition cycle ratio.

After post-annealing at 900 °C, the optical bandgaps of all of the films were increased, which is due to the fact that the GaN films were completely oxidized to N-doped Ga_2_O_3_ films and the defect density is reduced. The calculated bandgaps of the undoped Ga_2_O_3_, N-Ga_2_O_3_, N-Ga(12)Zn(1), N-Ga(6)Zn(1), and N-Ga(3)Zn(1) films after post-annealing are 5.03, 5.05 eV, 5.22 eV, 5.27 eV and 5.31 eV, respectively. In comparison to the undoped Ga_2_O_3_ film, the N- and Zn-doped samples have larger bandgaps. At the same time, the bandgap is enlarged with the increasing Zn concentration, which may be attributed to the Burstein–Moss effect [19,36].

**Figure 6:**
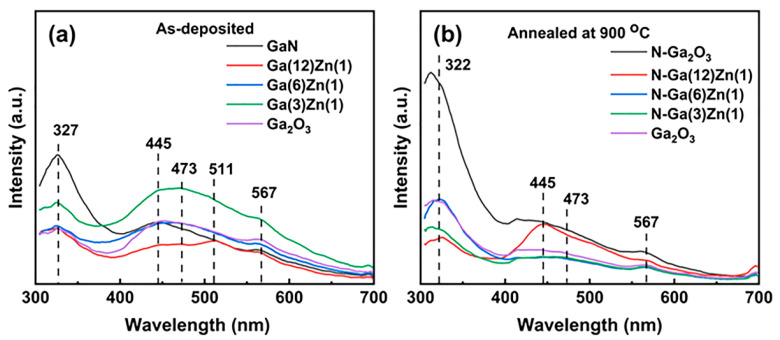
PL spectra of the as-deposited films before (**a**) and after (**b**) post-annealing at 900 °C.

The band structures and defect properties were investigated by photoluminescence spectroscopy, as demonstrated in Figure 6. Each of the samples shows a UV emission center around 327 nm, which can be attributed to the self-trapped excitons (STE). The blue emission peaks centered at 445 nm and 473 nm are considered to originate from the recombination of a trapped hole in an acceptor site with a trapped electron in a donor site, while the green emission peaks at 511 nm and 567 nm are caused by the neutral oxygen interstitial defects [37,38,39,40]. For the GaN, N-Ga(6)Zn(1), and Ga_2_O_3_ films, the intensity of the UV emission peak is strengthened after post-annealing, indicating that the number of non-radiative recombination centers are reduced [41]. As the numbers of donor defects and acceptor defects are suppressed after post-annealing, the blue emission of undoped Ga_2_O_3_ film is weakened [42]. However, the incorporation of N and Zn introduces an acceptor band into the N-Ga_2_O_3_ and N-Ga(12)Zn(1) films, and thereby the intensity of the blue emission peaks mounts up dramatically [23,43]. When the Zn doping concentration is raised further in the N-Ga(6)Zn(1) and N-Ga(3)Zn(1) films, the blue emission is suppressed due to the effect of concentration quenching [44]. After post-annealing, the intensity of green emission is reduced for all of the samples, suggesting that the quality of the films has been significantly enhanced.

## 4. Conclusions

In conclusion, Zn–N co-doped Ga_2_O_3_ films have been successfully grown on sapphire substrates using ALD followed with a post-annealing process. After being post-annealed at 900 °C, all of the films transform into a highly preferentially oriented β-Ga_2_O_3_, except for the sample of N-Ga(3)Zn(1), which remains amorphous. The HRTEM results reveal that an atomically sharp interface is formed and partial epitaxial growth takes place at the N-Ga(6)Zn(1) β-Ga_2_O_3_/α-Al_2_O_3_ interface. As the Zn doping concentration increases, the surface roughness of the films was reduced from 4.27 nm to 0.154 nm. In the visible wavelength region, all of the films exhibit an ultra-high optical transmittance, and the optical transmittance increases with the Zn doping concentration in the UV wavelength region. The bandgap of the Ga_2_O_3_ films can be adjusted from 5.07 eV to 5.41 eV by changing the Zn doping concentration. The PL spectra of the films exhibit several emission centers around 327 nm, 445 nm, 473 nm, 511 nm, and 567 nm under the excitation wavelength of 280 nm. A decline in the green luminescence was observed after post-annealing, demonstrating a decrease in oxygen interstitial defects. This work demonstrates that the structural and optical properties of β-Ga_2_O_3_ film can be well tuned by Zn–N co-doping and post-annealing. These findings facilitate the development of Ga_2_O_3_-based solar-blind ultraviolet photodetectors and other optoelectronic devices.

## Figures and Tables

**Table 1:** Detailed processing parameters for deposition of Ga_2_O_3_-based thin film under different conditions.

Sample	Growth Cycles of GaN	Growth Cycles of ZnO	Number of Supercycle	Growth Cycles of Ga_2_O_3_
GaN	500	0	/	/
Ga:Zn = 12:1	468	39	39 (12GaN + 1ZnO)	/
Ga:Zn = 6:1	450	75	75 (6GaN + 1ZnO)	/
Ga:Zn = 3:1	375	125	125 (3GaN + 1ZnO)	/
Ga_2_O_3_	/	/	/	500

## Data Availability

Data is contained within the article.

## References

[1] Alema F., Seryogin G., Osinsky A., Osinsky A. (2021). Ge doping of β-Ga_2_O_3_ by MOCVD. APL Mater..

[2] Chen Y.C., Yang X., Zhang C.Y., He G.H., Chen X.X., Qiao Q., Zang J.H., Dou W.J., Sun P.X., Deng Y. (2022). Ga_2_O_3_-Based Solar-Blind Position-Sensitive Detector for Noncontact Measurement and Optoelectronic Demodulation. Nano Lett..

[3] Lyle L.A.M., Back T.C., Bowers C.T., Green A.J., Chabak K.D., Dorsey D.L., Heller E.R., Porter L.M. (2021). Electrical and chemical analysis of Ti/Au contacts to β-Ga_2_O_3_. APL Mater..

[4] Li L., Li C., Wang S., Lu Q., Jia Y., Chen H. (2023). Preparation of Sn-doped Ga_2_O_3_ thin films and their solar-blind photoelectric detection performance. J. Semicond..

[5] Yan S., Yang G., He H., Liu Q., Peng Q., Chen J., Li M., Lu Y., He Y. (2023). High-Performance Self-Driven Solar-Blind Ultraviolet Photodetectors Based on HfZrO_2_/beta-Ga_2_O_3_ Heterojunctions. ACS Appl. Mater. Interfaces.

[6] Alema F., Hertog B., Ledyaev O., Volovik D., Thoma G., Miller R., Osinsky A., Mukhopadhyay P., Bakhshi S., Ali H. (2017). Solar blind photodetector based on epitaxial zinc doped Ga_2_O_3_ thin film. Phys. Status Solidi A.

[7] Ilhom S., Mohammad A., Shukla D., Grasso J., Willis B.G., Okyay A.K., Biyikli N. (2021). Low-Temperature As-Grown Crystalline β-Ga_2_O_3_ Films via Plasma-Enhanced Atomic Layer Deposition. ACS Appl. Mater. Interfaces.

[8] Wang C.L., Zhang J.C., Xu S.R., Zhang C.F., Feng Q., Zhang Y.C., Ning J., Zhao S.L., Zhou H., Hao Y. (2021). Progress in state-of-the-art technologies of Ga_2_O_3_ devices. J. Phys. D Appl. Phys..

[9] Wang Y.F., Su J., Lin Z.H., Zhang J.C., Chang J.J., Hao Y. (2022). Recent progress on the effects of impurities and defects on the properties of Ga_2_O_3_. J. Mater. Chem. C.

[10] Choi Y.H., Baik K.H., Kim S., Kim J. (2021). Photoelectrochemical etching of ultra-wide bandgap β-Ga_2_O_3_ semiconductor in phosphoric acid and its optoelectronic device application. Appl. Surf. Sci..

[11] Gupta C., Pasayat S.S. (2022). Vertical GaN and Vertical Ga_2_O_3_ Power Transistors: Status and Challenges. Phys. Status Solidi A.

[12] Wang J., Ji X., Qi S., Li Z., Yan Z., Li M., Yan X., Zhong A., Lu C., Qi X. (2023). Regulation of oxygen vacancies in nitrogen-doped Ga_2_O_3_ films for high-performance MSM solar-blind UV photodetectors. J. Mater. Chem. C.

[13] Kumarbekov K.K., Kakimov A.B., Karipbayev Z.T., Kassymzhanov M.T., Brik M.G., Ma C.-g., Piasecki M., Suchikova Y., Kemere M., Konuhova M. (2025). Temperature-dependent luminescence of europium-doped Ga_2_O_3_ ceramics. Opt. Mater. X.

[14] Luchechko A., Vasyltsiv V., Stasiv V., Kushlyk M., Kostyk L., Włodarczyk D., Zhydachevskyy Y. (2024). Luminescence spectroscopy of Cr3+ ions in bulk single crystalline β-Ga2O3-In2O3 solid solutions. Opt. Mater..

[15] Alema F., Itoh T., Vog S., Speck J.S., Osinsky A. (2022). Highly conductive epitaxial β-Ga_2_O_3_ and β-(Al_x_Ga_1−x_)_2_O_3_ films by MOCVD. Jpn. J. Appl. Phys..

[16] Hu D.Q., Wang Y., Zhuang S.W., Dong X., Zhang Y.T., Yin J.Z., Zhang B.L., Lv Y.J., Feng Z.H., Du G.T. (2018). Surface morphology evolution and optoelectronic properties of heteroepitaxial Si-doped β-Ga_2_O_3_ thin films grown by metal-organic chemical vapor deposition. Ceram. Int..

[17] Mi W., Du X.J., Luan C.N., Xiao H.D., Ma J. (2014). Electrical and optical characterizations of β-Ga_2_O_3_: Sn films deposited on MgO(110) substrate by MOCVD. RSC Adv..

[18] Zhang X.Y., Yang Y., Fan W.H., Wang C., Wu W.Y., Tseng M.C., Wuu D.S., Gao P., Kuo H.C., Lien S.Y. (2022). Growth and characterization of Si-doped Ga_2_O_3_ thin films by remote plasma atomic layer deposition: Toward UVC-LED application. Surf. Coat. Technol..

[19] Tao J.J., Lu H.L., Gu Y., Ma H.P., Li X., Chen J.X., Liu W.J., Zhang H., Feng J.J. (2019). Investigation of growth characteristics, compositions, and properties of atomic layer deposited amorphous Zn-doped Ga_2_O_3_ films. Appl. Surf. Sci..

[20] Galazka Z., Ganschow S., Fiedler A., Bertram R., Klimm D., Irmscher K., Schewski R., Pietsch M., Albrecht M., Bickermann M. (2018). Doping of Czochralski-grown bulk β-Ga_2_O_3_ single crystals with Cr, Ce and Al. J. Cryst. Growth.

[21] Feng Q.J., Liu J.Y., Yang Y.Q., Pan D.Z., Xing Y., Shi X.C., Xia X.C., Liang H.W. (2016). Catalytic growth and characterization of single crystalline Zn doped p-type β-Ga_2_O_3_ nanowires. J. Alloys Compd..

[22] Yan C.Y., Su J., Wang Y.F., Lin Z.H., Zhang J.C., Chang J.J., Hao Y. (2021). Reducing the acceptor levels of p-type β-Ga_2_O_3_ by (metal, N) co-doping approach. J. Alloys Compd..

[23] Su Y.L., Guo D.Y., Ye J.H., Zhao H.L., Wang Z., Wang S.L., Li P.G., Tang W.H. (2019). Deep level acceptors of Zn-Mg divalent ions dopants in β-Ga_2_O_3_ for the difficulty to p-type conductivity. J. Alloys Compd..

[24] Jiang Z.X., Wu Z.Y., Ma C.C., Deng J.N., Zhang H., Xu Y., Ye J.D., Fang Z.L., Zhang G.Q., Kang J.Y. (2020). P-type β-Ga_2_O_3_ metal-semiconductor-metal solar-blind photodetectors with extremely high responsivity and gain-bandwidth product. Mater. Today Phys..

[25] Wu Z.Y., Jiang Z.X., Ma C.C., Ruan W., Chen Y., Zhang H., Zhang G.Q., Fang Z.L., Kang J.Y., Zhang T.Y. (2021). Energy-driven multi-step structural phase transition mechanism to achieve high-quality p-type nitrogen-doped β-Ga_2_O_3_ films. Mater. Today Phys..

[26] Ardenghi A., Bierwagen O., Falkenstein A., Hoffmann G., Lähnemann J., Martin M., Mazzolini P. (2022). Toward controllable Si-doping in oxide molecular beam epitaxy using a solid SiO source: Application to β-Ga_2_O_3_. Appl. Phys. Lett..

[27] Azizie K., Hensling F.V.E., Gorsak C.A., Kim Y., Pieczulewski N.A., Dryden D.M., Senevirathna M.K.I., Coye S., Shang S.-L., Steele J. (2023). Silicon-doped β-Ga_2_O_3_ films grown at 1 µm/h by suboxide molecular-beam epitaxy. APL Mater..

[28] Wang C., Li S.W., Fan W.H., Zhang Y.C., Lin H.J., Zhang X.Y., Lien S.Y., Zhu W.Z., Wuu D.S. (2022). Annealing temperature controlled crystallization mechanism and properties of gallium oxide film in forming gas atmosphere. J. Am. Ceram. Soc..

[29] Zhang J., Wang W., Wu S., Geng Z., Zhang J., Chen L., Liu N., Yan X., Zhang W., Ye J. (2023). Exploratory phase stabilization in heteroepitaxial gallium oxide films by pulsed laser deposition. J. Alloys Compd..

[30] Zhang T., Li Y., Cheng Q., Hu Z., Ma J., Yao Y., Zuo Y., Feng Q., Zhang Y., Zhou H. (2022). Influence of O_2_ pulse on the β-Ga_2_O_3_ films deposited by pulsed MOCVD. Ceram. Int..

[31] Borujeny E.R., Sendetskyi O., Fleischauer M.D., Cadien K.C. (2020). Low Thermal Budget Heteroepitaxial Gallium Oxide Thin Films Enabled by Atomic Layer Deposition. ACS Appl. Mater. Interfaces.

[32] Liu X.T., Wang S.Q., He L., Jia Y.F., Lu Q., Chen H.F., Ma F., Hao Y. (2022). Growth characteristics and properties of Ga_2_O_3_ films fabricated by atomic layer deposition technique. J. Mater. Chem. C.

[33] Zhang L.Y., Yan J.L., Zhang Y.J., Li T., Ding X.W. (2012). A comparison of electronic structure and optical properties between N-doped β-Ga_2_O_3_ and N-Zn co-doped β-Ga_2_O_3_. Phys. B.

[34] Allen T.G., Cuevas A. (2015). Plasma enhanced atomic layer deposition of gallium oxide on crystalline silicon: Demonstration of surface passivation and negative interfacial charge. Phys. Status Solidi–Rapid Res. Lett..

[35] Kumar M., Poulose A.C., Nakajima Y., Sakthikumar D., Kumar V., Singh R. (2018). Anomalous emission from oxygen incorporated GaN nanowires. Phys. E.

[36] Saw K.G., Aznan N.M., Yam F.K., Ng S.S., Pung S.Y. (2015). New Insights on the Burstein-Moss Shift and Band Gap Narrowing in Indium-Doped Zinc Oxide Thin Films. PLoS ONE.

[37] Cooke J., Ghadbeigi L., Sun R.J., Bhattacharyya A., Wang Y.H., Scarpulla M.A., Krishnamoorthy S., Sensale-Rodriguez B. (2020). Synthesis and Characterization of Large-Area Nanometer-Thin β-Ga_2_O_3_ Films from Oxide Printing of Liquid Metal Gallium. Phys. Status Solidi A.

[38] Liu H., Xu C.X., Pan X.H., Ye Z.Z. (2020). The Photoluminescence Properties of β-Ga_2_O_3_ Thin Films. J. Electron. Mater..

[39] Ogugua S.N., Swart H.C., Ntwaeaborwa O.M. (2020). Effects of deposition environment and temperature on photoluminescence, particle morphology, and crystal structure of pulsed laser deposited Ga_2_O_3_ thin films. J. Vac. Sci. Technol. A.

[40] Shi Q., Wang Q.R., Zhang D., Wang Q.L., Li S.H., Wang W.J., Fan Q.L., Zhang J.Y. (2019). Structural, optical and photoluminescence properties of Ga_2_O_3_ thin films deposited by vacuum thermal evaporation. J. Lumin..

[41] Zhang T., Li Y., Zhang Y., Feng Q., Ning J., Zhang C., Zhang J., Hao Y. (2020). Effect of Temperature on the Structural and Optical Properties of Ga_2_O_3_ Thin Films Grown on m-plane Sapphire Substrates by Low-Pressure MOCVD. ECS J. Solid. State Sci. Technol..

[42] Mi W., Luan C.N., Li Z., Zhao C.S., Feng X.J., Ma J. (2013). Ultraviolet-green photoluminescence of β-Ga_2_O_3_ films deposited on MgAl_6_O_10_ (100) substrate. Opt. Mater..

[43] Yue W., Yan J., Wu J., Zhang L. (2012). Structural and optical properties of Zn-doped β-Ga_2_O_3_ films. J. Semicond..

[44] Li W.H., Peng Y.K., Wang C., Zhao X.L., Zhi Y.S., Yan H., Li L.H., Li P.G., Yang H.J., Wu Z.P. (2017). Structural, optical and photoluminescence properties of Pr-doped β-Ga_2_O_3_ thin films. J. Alloys Compd..

